# Building sensor coverage in couture: balancing cost, coverage, and comfort

**DOI:** 10.1038/s41598-025-21040-3

**Published:** 2025-10-23

**Authors:** Mahdi Ahmadnia, Mojtaba Maghrebi, Arezoo Shirazi, Alireza Ahmadian Fard Fini

**Affiliations:** 1 Department of Applied Mathematics, Faculty of Mathematics, Ferdowsi University of Mashhad, Mashhad, Islamic Republic of Iran; 2 Department of Civil Engineering, Faculty of Engineering, Ferdowsi University of Mashhad, Mashhad, Islamic Republic of Iran; 3 Department of Construction Management, California State University, Fresno, USA; 4 School of Built Environment, University of Technology Sydney, Sydney, Australia

**Keywords:** Sensor layout, HVAC system, Indoor air quality, Sensor placement, Optimization, Integer programming, Bilevel programming, Engineering, Mathematics and computing

## Abstract

The layout and performance of air quality sensors are crucial for building control systems and occupant comfort. Most previous studies have primarily focused on sensor placement and density. However, aspects such as accuracy, multifunctionality, and cost-effectiveness have received relatively little attention. To address these gaps, this study proposes two integer linear programming (ILP) models to optimize sensor placement. One model is designed to optimize cost, while the other focuses on maximizing coverage. To achieve an optimal balance between cost and coverage, a Leader-Follower (bi-level) approach is employed. This approach enables attaining the required coverage at the lowest possible cost or maximizing coverage within a fixed budget. Measurement accuracy at different locations is also incorporated to support energy efficiency and occupant satisfaction. The integration of the ILP models with the Leader-Follower framework reduces computational complexity and runtime while preserving solution accuracy. The results demonstrate that the proposed method can efficiently and rapidly identify optimal sensor configurations while ensuring both reliable coverage and cost-effectiveness.

## Introduction

Indoor air quality is a crucial aspect of occupant comfort in buildings^1,2^. Heating, Ventilation, and Air Conditioning (HVAC) systems are employed to create a comfortable indoor environment in office, commercial, and residential buildings, as well as workshops, laboratories, and industrial facilities^3,4^. The energy consumption of these systems accounts for a significant portion of a building’s electricity usage^5–10^. While inefficient HVAC operation leads to increased energy consumption, it can also reduce occupant satisfaction^10–13^. Efficient HVAC operation depends, among other factors, on sensory data. HVAC systems aim to maintain target indoor air quality with minimal energy consumption^14–16^. Therefore, the performance of climate control sensors significantly affects both the energy usage of HVAC systems and occupant satisfaction within buildings^17–19^. Sensors measuring temperature, humidity, air velocity, CO_2_, Particulate Matter (PM) and Volatile Organic Compounds (VOC), and in some cases, occupancy are used for the intelligent and efficient regulation of HVAC systems^20–22^. These sensors monitor the required parameters at various locations and transmit data to the HVAC control system^21,23^.

Sensor accuracy can vary significantly depending on sensor type^24–27^. Inaccurate measurements can compromise HVAC energy efficiency and reduce occupant satisfaction^27–30^. Therefore, deploying sensors with lower error rates is especially important in critical locations, such as crowded areas or zones monitored by a few sensors. Increasing the number of sensors can provide HVAC systems with more detailed and accurate information across different building areas, thereby improving their performance^16,31,32^. However, a larger number of sensors also increases procurement and installation costs, maintenance demands, data communication load, energy consumption, memory storage requirements, and may contribute to user discomfort^33–38^.

Virtual sensing, i.e. estimating data for a location without a sensor by interpolating or extrapolating information from physical sensors installed elsewhere, is a cost-effective and efficient solution to these challenges^39–46^. In addition, virtual sensing can be used for error correction in physical sensors and the detection of sensor malfunctions^39,42,47^. The accuracy of virtual sensing depends on a building’s specific conditions, including the type and location of heating and cooling systems, heat sources, occupancy levels, window positions (open or closed), and sensor placement^14,40,47,48^. For example, virtual sensing based on sensors placed near extreme temperature zones (e.g., adjacent to heating or cooling systems) typically results in higher error rates. Besides, buildings with significant temperature fluctuations or multiple heating and cooling systems generally require more sensors for accurate virtual sensing^6,45,49^. Therefore, carefully determining the number, type (accuracy), and placement of sensors, while accounting for the unique conditions of each building, can enhance virtual sensing accuracy and in turn, improve HVAC system performance^18^.

While some physical sensors, such as contact sensors, measure parameters only at the point of installation, non-contact sensors (e.g. those using infrared or laser technology) can cover a certain radius and provide similar measurements^50^. Compared to contact sensors, non-contact sensors can reduce the number of sensors required in a building, as they are capable of monitoring multiple temperature conditions from a single location. However, non-contact sensors typically involve higher procurement costs and may introduce greater measurement errors than contact sensors. Therefore, choosing between deploying non-contact sensors or increasing the number of contact sensors is a critical decision that significantly impacts HVAC system performance.

The accuracy of virtual sensing and non-contact sensors typically decreases with distance from the installation point. Placing sensors closer to occupant areas can enhance energy efficiency and comfort^5^. High-occupancy zones such as conference rooms and restaurants benefit from denser sensor placement and higher accuracy sensors^14,51,52^. In dynamic environments—like universities, offices, and seminar rooms—occupancy levels vary over time^53–55^. While historical data can help estimate presence, these predictions are prone to errors due to factors like schedule changes or staff absences^45,56^. Employing occupancy sensors in these locations can provide a more accurate and reliable solution^7,57–59^. Therefore, HVAC systems can prioritize crowded areas to improve occupant satisfaction and avoid excessive energy consumption in unoccupied areas^1,3,54^,^59–61^.

Although HVAC systems rely on temperature, humidity, and air velocity data from various points within a building to maintain a comfortable indoor environment, these parameters do not equally impact system performance, temperature is the most critical, followed by humidity and air velocity^62,63^. As a result, the number and distribution of these sensors are typically unequal^64^. Additionally, some sensors are multifunctional, capable of measuring two or more parameters simultaneously^64^. While such sensors can reduce total sensor count, lower procurement costs, and improve building aesthetics, they often come at the expense of higher unit costs and potentially lower measurement accuracy. Balancing these trade-offs, sensor functionality, cost, accuracy, and coverage, is a complex problem. A systematic method is required to design an optimal sensor layout that considers these conflicting factors. Integer Linear Programming (ILP) offers a powerful tool for solving such optimization problems through providing exact solutions that can minimize cost while maximizing system performance. Despite the applicability of ILP models to such a case, existing research has not applied ILP to the problem of sensor placement in HVAC systems. Moreover, prior studies have largely overlooked the role of sensor accuracy, multifunctionality, and cost variation in determining optimal sensor layouts.

To address this gap, this paper introduces two ILP models that jointly determine the type, number, and placement of sensors. A Leader-Follower approach is employed to integrate and solve these models simultaneously. The proposed method explicitly incorporates varying sensor accuracies, multifunctional capabilities, and cost differences. It aims to maximize coverage based on measurement accuracy and the importance of monitored locations, all while minimizing overall cost. This optimization can lead to improved energy efficiency and enhanced occupant satisfaction, offering a practical and scalable solution to sensor deployment in HVAC systems. The proposed approach can ensure maximum coverage at the minimum cost. Coverage optimization is based on the measurement accuracy of the covered locations and the importance of those points. This can lead to increased energy efficiency and occupant satisfaction.

## Literature review

Various studies have been conducted to identify the factors influencing optimal sensor layout. Among these, works like^10,29,39,65^, have investigated the impact of the error of physical sensors and virtual sensors (virtual sensing) on HVAC systems. Other relevant studies, such as^59^, ^66–68^, have demonstrated that accounting for both the location and number of occupants can significantly improve the energy efficiency of HVAC systems and enhance occupant satisfaction. Hence, the type (accuracy), number, and location of sensors can influence the performance of HVAC systems.

Sensor layout optimization has received significant attention in the literature. Ploennigs et al.^69^ used scalable simple model-based virtual sensors to analyze the heat consumption of buildings using underfloor heating. Chen and Li^70^ introduced a Bayesian model framework in designing a virtual sensor by minimizing the amount of physical sensors. Kondo et al.^45^ used equation-based modeling and optimization-based parameter estimation to enhance the accuracy and handle virtual sensing uncertainties. To address various sources of uncertainty - such as precision degradation, systematic bias, temporal drift, measurement noise, and environmental influences - Wang et al.^27^ applied Bayesian theory and Markov Chain Monte Carlo methods. The approach of calibrating virtual sensors to reduce errors was highlighted by Yoon et al.^71^ and Choi et al.^72^. To simultaneously increase the speed and accuracy of virtual sensing, Edtmayer et al.^43^ developed a digital twin model that includes building energy simulation and Computational Fluid Dynamics (CFD). In this approach, the mean absolute error was 0.35 K for temperature and 1.2% for humidity. Oliver and Sarkar^31^ trained a supervisory framework for virtual sensing of temperature and humidity using data collected from temporary sensors over a month. This supervisory framework reduced the number of sensors during the operational period and by doing so, achieved a 60–80% reduction in the cost of building sensor network.

Du et al.^73^ by integrating Building Energy Simulation (BES) and CFD, showed that the installation location of sensors significantly impacts the energy efficiency of HVAC systems and occupant satisfaction. Similarly, Tian et al.^48^ showed the impact of sensor location on the performance of HVAC systems by combining BES with GenOpt. Inspired by fractal theory, Xie et al.^47^ have proposed an algorithm for locating sensors to improve the accuracy of virtual sensing. Nguyen et al.^37^ have addressed the problem of reducing sensor cost and virtual sensing error in optimal sensor placement by proposing a greedy algorithm. In order to minimize the uncertainties in virtual sensing, Nguyen et al.^74^ have used Gaussian processes for sensor location determination. Chen and Gorlé^75^ attempted to enhance the measurement precision by quantifying uncertainties and optimizing the temperature sensor location.

Shin and Kwak^44^ focused on the performance of virtual sensors in determining the location of physical sensors. Their method resulted in an error rate of below 2% across various scenarios. Kowli et al.^14^ calculated the point-wise correlation in their approach that makes use of the Pearson correlation coefficient, making it easier to discern which points may be used optimally for deployment. This approach achieved an absolute error of less than 0.6 °C in 93% of cases and less than 1 °C in 99.6% of cases. Data-driven approaches for sensor number and placement optimization have also been proposed by Yoganathan et al.^16^ and Suryanarayanaet al.^32^. Yoganathan et al. applied clustering algorithms and the Pareto principle to develop optimum sensor location schemes, whereas Suryanarayana et al.^32^ utilized statistical methods for developing sensor correlation that would help in removing some redundant sensors. Bucarelli and El-Gohary^17,49^ have made use of the clustering techniques in order to determine the number and locations of sensors. Sensor configuration in buildings was shown in^49^, to be able to influence the energy consumption predictions between 35% − 76%.

The optimal placement of sensors, through improved monitoring of indoor air quality, can play a significant role in enhancing occupants’ comfort and reducing energy consumption. In this regard, the optimal adjustment of environmental parameters related to thermal conditions and air quality control is of particular importance. For example, Verma et al.^76^ proposed a building management and information system based on a multi-agent topology, aiming to minimize the discrepancy between the actual environmental conditions and the desired comfort conditions. In a similar study^77^, they developed a machine learning-based controller to reduce the gap between the user-defined setpoint temperature and the actual indoor temperature. Verma et al.^78^ employed the particle swarm optimization algorithm to determine the optimal values of environmental parameters such as temperature, illumination level, and CO2 concentration according to user preferences. Extending this line of research, Maurya et al.^79^ applied a gradient-free optimization algorithm to include relative humidity in the set of optimized parameters.

The problem of sensor placement for HVAC control and management has several unique characteristics that sets it apart from the classical sensor placement problem in distributed sensor networks. The most important differences are summarized as follows:

- **Multi-objective nature of the problem**: In classical formulations, the primary aim is typically the minimization of deployment costs or the number of sensors. In contrast, HVAC systems require not only cost minimization but also the enhancement of occupants’ thermal comfort and indoor environmental quality. These two goals are often contradictory and require a two-level framework such as leader-follower to balance them.
- **Different coverage requirements**: In distributed sensor networks, full coverage of the monitored region is usually considered mandatory, and all locations are given equal importance. In contrast, in HVAC systems, there is no requirement for 100% coverage and control objectives may be achieved with partial coverage. Moreover, the importance of different zones (e.g., frequently occupied versus rarely occupied rooms) is not uniform and must be weighted accordingly in the model.
- **Multi-parametric measurements**: While many classical studies consider the monitoring of a single parameter (e.g., target detection or signal intensity), HVAC systems require the simultaneous measurement of multiple parameters such as temperature, humidity, CO₂ concentration, and pollutants. The use of multifunctional sensors and the varying importance of these parameters for occupants’ comfort and health significantly increase the complexity of the placement problem.

For these reasons, existing approaches in distributed sensor networks (such as^80–83^ may share methodological elements but are insufficient to address the specific challenges of HVAC sensor placement. Therefore, it is necessary to investigate this problem independently, considering all the unique factors.

The research that has gone on to date towards optimizing sensor layout in buildings to increase efficiency in energy consumption, measurement accuracy, and spatial coverage is generally heuristic or rule-based, geometrical, or done by trial-and-error methods. However, critical factors such as differences in sensor accuracy, the availability of multifunctional sensors, and sensor purchase and maintenance costs have received less attention in the extant literature. These are important factors because sensors differ significantly in their precision, reliability, and ability to measure multiple environmental parameters. Thus, this directly influences sensor cost-effectiveness and performance.

In addition, previous works have overlooked the use of exact optimization methods like integer programming, in solving the sensor layout problem for HVAC systems. Integer programming is a systematic, quantitative method of making decisions, which is especially appropriate for this problem because of its inherently discrete and combinatorial nature. By incorporating constraints like type, cost, and accuracy of the sensor, as well as spatial and operational requirements, integer programming is able to provide an exact optimal solution that heuristic methods may fail to achieve.

Considering the complexity of the decision-making process in the sensor layout problem, this paper uses integer programming to optimally identify the number, type, and location of sensors. The introduced approach in this paper tries to minimize the cost of sensors with the purpose of enhancing measurement accuracy and increasing target area coverage. In order to enhance the practical applicability of the proposed approach, a weighting system is adopted that will allow priority in sensor layout for more critical or densely occupied areas such as high-traffic zones, conference rooms, or shared spaces. Larger weights will therefore be associated with these areas to make sure that accurate measurement of environmental quantities is made where most needed, reducing overall energy consumption by avoiding sensor redundancy in less significant locations. This enhances occupant comfort and satisfaction while mitigating energy waste, thereby aligning with broader goals of sustainability and operational efficiency in building management systems.

## Methodology

ILP is a mathematical optimization technique in which decision variables must take integer values. It has been one of the most widely used approaches for solving equipment layout and location optimization problems. ILP is well-suited to handling multiple and often conflicting objectives as it allows for the incorporation of specific and complex constraints that are unique to a given problem. One of the key advantages of ILP is its ability to produce precise optimal solutions, which can significantly impact cost, performance, and efficiency.

Given these strengths, ILP can be effectively applied to sensor layout problems, where multiple objectives, such as minimizing sensor costs, maximizing measurement accuracy, and ensuring optimal coverage of target areas, must be considered simultaneously. However, ILP methods have not yet been widely explored in the context of sensor layout optimization. In this paper, ILP is applied with the aim of addressing multiple objectives while achieving precise and optimal solutions.

In this study, the sensor placement problem is formulated as a *bi-level programming* problem and solved using a *Leader-Follower Approach*. The two concepts are defined as follows:

### Bi-level programming

A hierarchical optimization framework consisting of two decision-making levels.

### Leader-Follower approach

A specific type of bi-level programming in which the upper level (leader) makes a decision first, and the lower level (follower) makes its decision based on the leader’s choice.

Accordingly, two distinct ILP models are formulated to optimize cost and coverage.

- *Cost Optimization Model*: This model aims to achieve full environmental coverage at the minimum possible cost.
- *Coverage Optimization Model*: This model intends to maximize environmental coverage within a predefined budget.

The proposed Leader-Follower approach solves these two models sequentially, thereby achieving an optimal balance between cost and coverage. The methodology is developed based on the following assumptions and criteria:

- The entire environment is divided into small blocks.
- Candidate locations for sensor installation are predetermined.
- The number, location, and type of required sensors are determined through the optimization process.
- Contact sensors can only measure the installation point, whereas non-contact sensors can cover a specific radius.
- Sensors differ in terms of measurement accuracy, coverage radius, and cost.
- The importance of covering each block can vary. Consequently, a weight is assigned to each block to reflect its level of significance (as an input parameter).
- Some blocks are measured by physical sensors, while others are measured by virtual sensors.
- If a block is measured by multiple physical or virtual sensors, the most accurate value is assigned to that block.
- Various parameters such as temperature, humidity, and air velocity may need to be measured. The importance of measuring each parameter can vary.
- Each sensor can measure one or multiple parameters simultaneously. The accuracy of a sensor in measuring one parameter may differ from its accuracy in measuring another parameter.
- In a block, the virtual measurement accuracy of one parameter can differ from that of another parameter.
- Environmental coverage is defined as the weighted average of the measurement accuracy of parameters across all blocks, where the weight of each block reflects its importance.

**Fig. 1:**
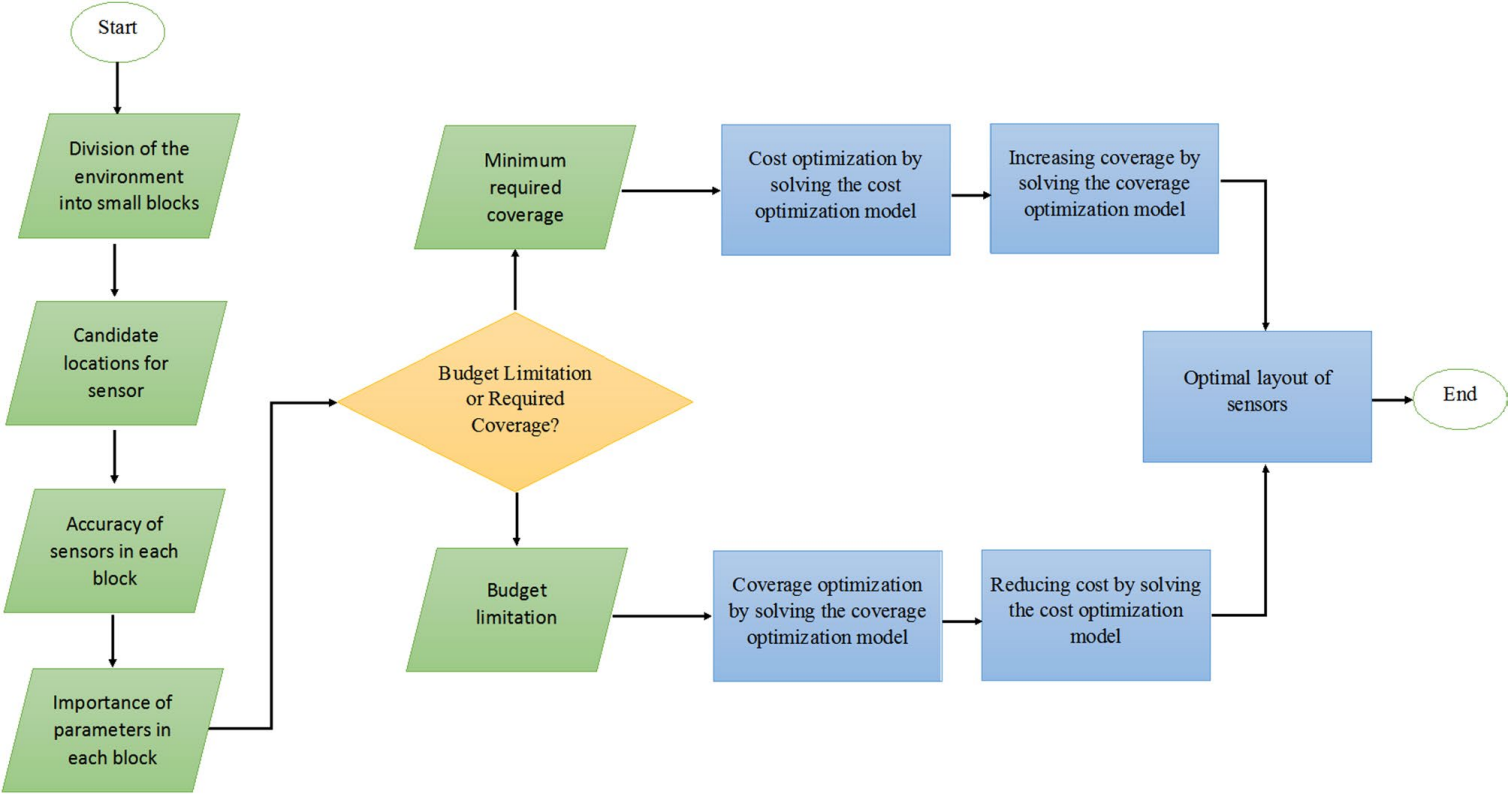
Flowchart of the proposed method.

Figure 1 illustrates the flowchart of the proposed method. As shown, the process begins with dividing the entire environment into small blocks. Candidate locations for sensor placement are then identified. Two key parameters of the proposed approach are considered: the measurement accuracy within each block and the importance assigned to each block. Next, the desired strategy is determined. If financial constraints are prioritized, the *Budget Constraint Strategy* is selected; whereas if a specific coverage target is intended, the *Minimum Coverage Assurance Strategy* is applied.

In the case of the *Minimum Coverage Assurance Strategy*, the cost optimization model is first solved to determine the optimal budget. Subsequently, the coverage optimization model is applied to maximize environmental coverage while maintaining the optimal cost. Conversely, in the *Budget Constraint Strategy*, the coverage optimization model is solved first to identify the maximum achievable coverage. This is then followed by the cost optimization model, which minimizes expenses while preserving the achieved coverage.

The description of the parameters and variables of the proposed ILPs are listed in Table 1.

**Table 1:** Description of the parameters and variables of the proposed model.

Indexes	Description
*Sets*	
$\:K$	Set of all types of sensors available (index $\:k$).
$\:L$	Set of candidate locations for sensor installation (index $\:l$).
$\:P$	Set of all the blocks of the desired environment (index $\:p$).
$\:T$	Set of all parameters to be measured (index $\:t$)
*Parameters*	
$\:{C}_{k}$	The cost of purchasing and installing the sensor of type $\:k$.
$\:{\lambda\:}_{klpt}$	The measurement accuracy of sensor $\:k$ installed at location $\:l$ to measure parameter $\:t$ in block $\:p$.
$\:{w}_{pt}$	It is a weight that shows the importance of measuring parameter $\:t$ in block $\:p$.
$\:SW$	The sum of the weights of all blocks.
$\:\stackrel{-}{Cover}$	Lower bound to cover the entire environment
$\:\stackrel{-}{Cost}$	Budget limit
$\:{LA}_{pt}$	lower bound of the measurement accuracy of the parameter t for the block p.
*Variables*	
$\:{x}_{kl}$	1 if sensor of type $\:k$ is installed at location $\:l$, 0 if otherwise.
$\:{y}_{klpt}$	1 if parameter $\:t$ in block $\:p$ is measured by the sensor of type $\:k$ installed in location l, and 0 if otherwise.
$\:Cove{r}_{pt}$	The measurement accuracy of parameter $\:t$ in block $\:p$

### Preprocessing

First, the whole environment is divided into small blocks. While several essential parameters should be measured within each block, such as temperature, humidity, and air velocity, the importance of measuring these parameters can vary across blocks. Factors such as the presence and location of residents, as well as the number of individuals in each block, influence the significance of each measurement^5,7,59^. Additionally, monitoring certain areas can impact energy efficiency and help identify undesirable behaviors, such as residents leaving doors or windows open, that may compromise environmental control^11,84^. Therefore, measuring specific parameters in some blocks may be of greater importance than in others. Moreover, the relative importance of temperature, humidity, and air velocity can differ even within the same block. Accordingly, one of the key inputs to the proposed method is the importance weight associated with measuring each parameter in each block. This is represented by $\:{w}_{pt}$, which denotes the weight indicating the importance of measuring parameter $\:t$ in block $\:p$.

When a sensor is installed in a block, it can directly measure parameters such as temperature, humidity, and air velocity in that specific block. In addition, it can provide *virtual measurements* for adjacent blocks. The accuracy of these measurements depends on several factors, including the type of sensor used, the characteristics of the environment, and the method employed for virtual estimation. Another key input to the proposed method is the measurement accuracy for each parameter in each block, assuming a sensor is installed nearby. This parameter must be calculated before the optimization process begins. This is denoted by $\:{\lambda\:}_{klpt},$ which represents the accuracy of measuring parameter of parameter $\:t$ in block $\:p$ when a sensor of type $\:k$ is installed at location $\:l$.

Based on this input data, the significance of each block, the importance of each parameter, and the measurement accuracy, the proposed method determines the optimal sensor layout, including the number, type, and placement of sensors.

### Cost optimization model

In this section, an ILP model is developed to ensure the desired coverage at the lowest possible cost. The model treats the minimum required coverage ($\:\stackrel{-}{Cover}$) as an input parameter, while the type, number, and location of sensors are considered as decision variables.

1 $$\:{min}z=\sum\:_{k\in\:K}\sum\:_{l\in\:L}{C}_{k}\times\:{x}_{kl}$$

Subject to:

2 $$\:\sum\:_{k\in\:K}{x}_{kl}\le\:1\:\:\:\:\:\:\:\:\:\:\:\:\:\:\:\:\:\:\:\:\:\:\:\forall\:l\in\:L$$

3 $$\:{y}_{klpt}\le\:{x}_{kl}\:\:\:\:\:\:\:\:\:\:\:\:\:\:\:\:\:\:\:\forall\:k\in\:K,\:\forall\:l\in\:L,\:\forall\:p\in\:P,\:\forall\:t\in\:T$$

4 $$\:\sum\:_{k\in\:K}\sum\:_{l\in\:L}{y}_{klpt}\le\:1\:\:\:\:\:\:\:\:\:\:\forall\:p\in\:P,\forall\:t\in\:T$$

5 $$\:Cove{r}_{pt}=\sum\:_{k\in\:K}\sum\:_{l\in\:L}{y}_{klpt}\times\:{\lambda\:}_{klpt}\:\:\:\:\:\:\:\:\:\:\:\forall\:p\in\:P,\:\forall\:t\in\:T$$

6 $$\:\frac{1}{SW}\times\:\sum\:_{p\in\:P}{W}_{pt}\times\:Cove{r}_{pt}\ge\:\stackrel{-}{Cover}\:\:\:\:\:\:\:\:\:\:\:\:\:$$

7 $$\:Cove{r}_{pt}\ge\:{LA}_{pt}\:\:\:\:\:\:\:\:\:\forall\:p\in\:P,\:\forall\:t\in\:T$$

Eq. (1) expresses the objective function that minimizes the cost of sensors. $\:{C}_{k}$ in Eq. (1) indicates the cost of buying and installing a sensor of type $\:k$. Also, $\:{x}_{kl}$ is a binary variable that is equal to 1 if a sensor of type $\:k$ is installed at location $\:l$ and equal to 0 otherwise. Accordingly, if a sensor is installed at a given location ($\:{x}_{kl}=1$), its associated cost is incorporated into the objective function.

Constraint (2) is added to the proposed model to avoid installing more than one sensor in one location. This constraint can be removed according to the specific needs of a building.

In Constraints (3) to (5), $\:{y}_{klpt}$ is a binary variable that takes the value 1 if parameter $\:t$ in block $\:p$ is measured by a sensor of type $\:k$ installed at location $\:l$, and 0 otherwise. If a sensor of type $\:k$ is not installed at location $\:l$ ($\:{x}_{kl}=0$), then parameter $\:t$ in block $\:p$ cannot be measured by that sensor ($\:{\lambda\:}_{klpt}=0$). Constraint (3) prevents the erroneous assignment of $\:{\lambda\:}_{klpt}=1\:$when the corresponding sensor is absent. Since some blocks may be measurable by multiple sensors, Constraint (4) ensures that only the reading of one sensor is considered for parameter $\:t$ in block $\:p$. Constraint (5) then calculates the measurement accuracy (coverage) of parameter $\:t$ in block $\:p$ ($\:Cove{r}_{pt}$), based on the selected sensor. Here, $\:{\lambda\:}_{klpt}$ denotes the measurement accuracy of sensor $\:k$ at location $\:l$ in measuring parameter $\:t$ for block $\:p$. Accordingly, the multiplication of $\:{\lambda\:}_{klpt}$ and $\:{y}_{klpt}$ yields the measurement accuracy (coverage) of parameter $\:t$ in block $\:p$. Therefore, the coverage rate in this paper refers to the measurement accuracy. Specifically, if a sensor covers a block, the coverage in that block is equal to the measurement accuracy of the sensor in that block. Conversely, if no sensor covers a block, the measurement accuracy in that block is considered zero.

Constraint (6) ensures that the desired minimum coverage is satisfied. $\:\stackrel{-}{Cover}\:$in Constraint (6) indicates the minimum required coverage of the entire environment. Also, $\:{w}_{pt}$ is a weight that shows the importance of measuring parameter t in block p. In Constraint (6), $\:SW$ represents the sum of the weights of all blocks. Constraint (6) calculates the overall coverage based on the weight of each block.

If specific coverage (measurement accuracy) is required for certain blocks, Constraint (7) ensures this. Where $\:{LA}_{pt}$ represents the lower bound of the measurement accuracy of the parameter t for the block p.

### Coverage optimization model

In this section, an ILP model is developed to maximize coverage while considering budget constraints. The model treats the budget constraint ($\:\stackrel{-}{Cost}$) as an input parameter, while the type, number, and location of sensors are considered decision variables.

8 $$max\;z = \frac{1}{{SW}} \times \:\sum\limits_{{p \in \:P}} {\sum\limits_{{t \in \:T}} {W_{{pt}} \times \:Cover_{{pt}} } }$$

Subject to:

9 $$\:{y}_{klpt}\le\:{x}_{kl}\:\:\:\:\:\:\:\:\:\:\:\:\:\:\:\:\:\:\:\forall\:k\in\:K,\:\forall\:l\in\:L,\:\forall\:p\in\:P$$

10 $$\sum\limits_{{k \in \:K}} {\sum\limits_{{l \in \:L}} {y_{{klpt}} \le \:1\:\:\:\:\:\:\:\:\:\:\forall \:p \in \:P} }$$

11 $$\:\:\sum\limits_{{_{{k \in \:K}} }} {x_{{kl}} \le \:1\:\:\:\:\:\:\:\:\forall \:l \in \:L}$$

12 $$Cover_{{pt}} = \sum\limits_{{k \in \:K}} {\sum\limits_{{_{{l \in \:L}} }} {y_{{klpt}} \times \:\lambda \:_{{klpt}} \:\:\:\:\:\:\:\:\:\:\:\forall \:p \in \:P} }$$

13 $$\:Cove{r}_{pt}\ge\:{LA}_{pt}\:\:\:\:\:\:\:\:\:\forall\:p\in\:P,\:\forall\:t\in\:T$$

14 $$Cost = \sum\limits_{{_{{k \in \:K}} }} {\sum\limits_{{_{{l \in \:L}} }} {C_{k} \times \:x_{{kl}} } }$$

15 $$Cost \le \:\:\overline{{Cost}}$$

Eq. (8) represents the objective function, optimizing the total coverage of the environment based on the importance of blocks and measurement parameters. The model aims to maximize the weighted average of measurement accuracy across all blocks within the given budget. Constraints (9) to (13) are identical to the Constraints of the *Cost Optimization Model*. Constraint (14) calculates the cost of the installed sensors, while Constraint (15) ensures the budget constraint.

### proposed leader-follower approach

The *Cost Optimization Model* is proposed to minimize cost, while the *Coverage Optimization Model* is designed to enhance measurement accuracy and maximize environmental coverage. To simultaneously optimize both cost and coverage, the *Leader-Follower Approach* (*Bilevel Programming*) is employed to integrate these two models. In this approach, one model is considered the leader and the other the follower. The leader model is solved first, and based on its results, the follower model is then solved. This process continues iteratively until an optimal solution is reached.

Based on the constraints and objectives, two strategies are proposed for employing the *Leader-Follower Approach* to solve the sensor layout problem: (1) *Minimum Coverage Assurance Strategy*, and (2) *Budget Constraint Strategy*. These two strategies are introduced in detail below. Finally, it is proven that both strategies converge in just one iteration.

### Minimum coverage assurance strategy

In this strategy, the *Cost Optimization Model* is used for decision-making at the Leader Level, while the *Coverage Optimization Model* is employed at the Follower Level. Accordingly, decisions at the Leader Level ensure the minimum required coverage at the lowest possible cost. At the Follower Level, without increasing the cost determined by the leader, the coverage (measurement accuracy) is maximized as much as possible. This strategy is summarized in *Algorithm 1*.


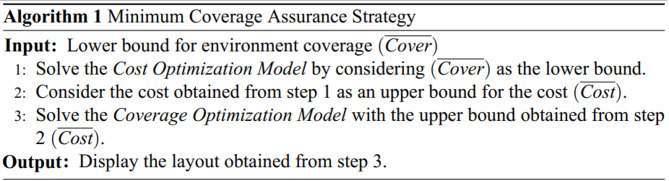


### Budget constraint strategy

In this strategy, the *Coverage Optimization Model* is used for decision-making at the Leader Level, while the *Cost Optimization Model* is employed at the Follower Level. Accordingly, the leader level aims to achieve the maximum possible coverage within the budget constraints. Subsequently, at the follower level, decisions are made to minimize costs as much as possible without reducing the coverage determined by the leader. This strategy is summarized in *Algorithm 2*.


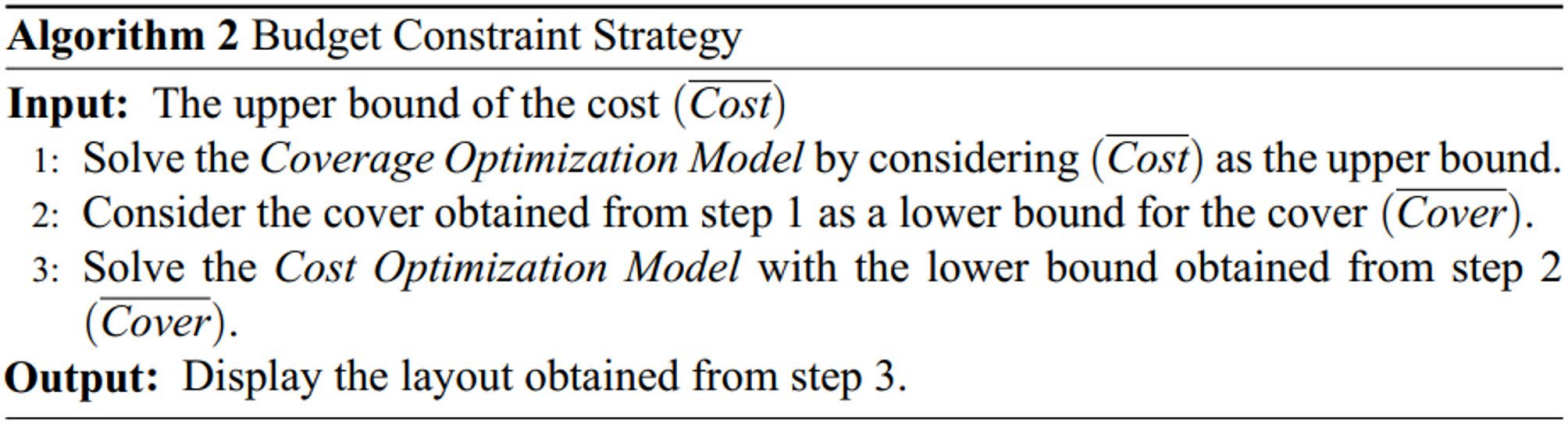


### convergence proof

In this section, the convergence of the proposed *Leader-Follower Approach* is proven for both introduced strategies.

**Convergence of the Minimum coverage assurance strategy:**

Suppose ($\:\stackrel{-}{Cover}$) is the lower bound of the cover and $\:{A}_{1}\:$is the cost obtained from the *Cost Optimization Model* in the first iteration. Also, $\:{B}_{1}\:$is the obtained coverage from the *Coverage Optimization Model* in the first iteration. Because $\:{A}_{1}$ is considered the upper bound of the *Coverage Optimization Model* ($\:{A}_{1}=\stackrel{-}{Cost}$) and the rest of the constraints of the two models are similar, the optimal solution of the *Cost Optimization Model* is a feasible solution for the *Coverage Optimization Model*. Therefore, $\:{B}_{1}\ge\:\stackrel{-}{Cover}$ (according to Constraint 6 and *Eq. 8*). In the second iteration, the *Cost Optimization Model* with the lower bound $\:{B}_{1}$ should be solved (In Constraint 6). Because $\:{B}_{1}\ge\:\stackrel{-}{Cover}\:$the solution space of the *Cost Optimization Model* becomes smaller in the second iteration compared to the first iteration. So, the optimal solution of the *Cost Optimization Model* does not improve in the second iteration ($\:{A}_{1}\le\:{A}_{2}$). Therefore, the algorithm reaches the optimal solution in the first iteration.

**Convergence of the Budget constraint strategy:**

The convergence proof of this strategy is similar to the *Minimum Coverage Assurance Strategy*.

### Computational complexity

The computational complexity of both strategies is related to step 1 and step 3. Specifically, the computational complexity of the proposed method is equal to the cumulative computational complexities of the *Cost Optimization Model* and *Coverage Optimization Models*. Both models are characterized as ILP models.

### Grouping of similar-usage floors in multi-story buildings

In multi-story buildings with diverse functional layouts, the number of variables and constraints in ILP-based sensor placement models may increase substantially. This expansion often results in higher memory requirements and longer computational times. This paper proposes an approximate technique (Grouping of Similar-Usage Floors) to address this challenge.

In many buildings, several floors share similar functional usage and architectural design. In such cases, only one of the similar floors is selected as a representative candidate, and the obtained placement configuration is generalized to all corresponding floors. This approach effectively reduces the number of variables and constraints in the model. To implement this method, the following modifications are introduced:

i. The weight of each block ($\:{W}_{pt}$) in the representative floor is multiplied by the number of similar floors. The total weight (SW) remains unchanged.
ii. The cost calculated for the representative floor is multiplied by the number of similar floors. Accordingly, the cost function is modified as follows:

$$\:Cost=\sum\:_{r\in\:R\:}\sum\:_{k\in\:K}\sum\:_{l\in\:{L}^{r}}{N{R}_{r}\times\:C}_{k}\times\:{x}_{kl}\:$$

where $\:R$ denotes the set of representative floors, and $\:N{R}_{r}$ is the number of floors similar to representative floor $\:r$. The set $\:{L}^{r}$ represents the candidate locations for sensor placement in representative floor $\:\:r.$.

## Case study

**Fig. 2:**
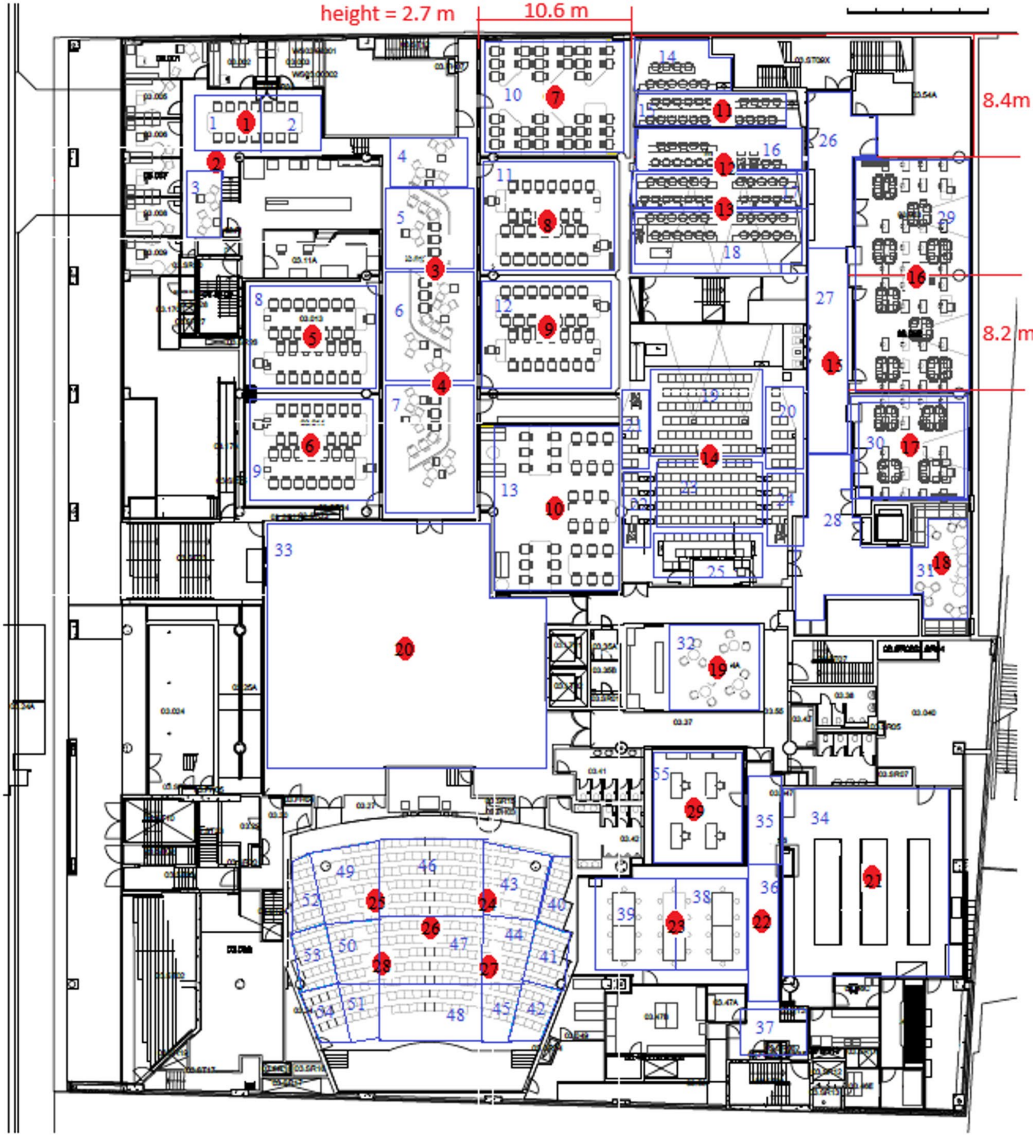
Floor Layout of the Case Study Area.

In this study, the testbed was located on a single floor of a university campus in Sydney, Australia. The selected area includes different indoor spaces such as classrooms, computer labs, and lecture theaters. The layout of the floor is shown in Fig. 2. This figure depicts the case study and shows, by red circles, the candidate locations for sensor installation. In total, 29 candidate locations for sensor installation have been selected. This figure further illustrates that the environment is divided into 55 small blocks, in blue. Regions that have similar conditions in terms of temperature, humidity, and air velocity are grouped into a single block.

The importance of covering the blocks shown in Fig. 2 varies based on the number of occupants, their duration of stay, the type of usage, and their share of energy consumption. Table 2 specifies the importance of measuring temperature, humidity, and air velocity parameters in each block ($\:{W}_{pt}$), based on expert opinions, by assigning values from 1 to 20.

**Table 2:** The importance of covering the blocks in Fig. 2.

Location	Temperature	Humidity	air velocity	Location	Temperature	Humidity	air velocity
1	4	1	3	29	14	8	2
2	3	1	3	30	7	4	1
3	3	1	3	31	10	2	2
4	3	1	3	32	8	3	3
5	5	1	3	33	8	2	10
6	6	1	3	34	4	1	0
7	6	1	3	35	2	0	2
8	20	10	4	36	2	1	3
9	20	10	4	37	2	0	2
10	20	10	4	38	4	1	3
11	20	10	4	39	4	1	3
12	20	10	4	40	4	2	1
13	20	10	4	41	6	4	2
14	8	5	2	42	5	3	2
15	16	10	4	43	6	5	2
16	16	10	4	44	6	5	2
17	16	10	4	45	4	2	1
18	16	10	4	46	18	10	4
19	18	10	4	47	18	10	4
20	4	2	1	48	8	5	2
21	2	1	0	49	6	4	2
22	2	1	0	50	6	4	2
23	17	9	4	51	4	2	1
24	4	2	1	52	4	2	1
25	10	5	2	53	6	4	2
26	2	0	3	54	5	3	2
27	2	0	3	55	2	0	2
28	2	0	3	**-**	-	-	-

Nine different types of sensors have been considered for installation at the candidate locations. These sensors measure air temperature, humidity, and air velocity. Simple sensors measure only one parameter, while multifunctional sensors can simultaneously measure two or three parameters. The details of the sensors, including their measurement accuracy and purchase price, are reported in Table 3.

**Table 3:** Details of candidate sensors for installation.

Sensor	Type	Measurement parameter (accuracy)			Cost ($)
		Temperature	Humidity	Velocity	
1	Simple non-contact	98%	–	–	400
2	Simple non–contact	97%	–	–	300
3	Simple contact	98%	–	–	200
4	Simple contact	97%	–	–	150
5	Simple contact	–	97%	–	300
6	Simple contact	–	–	95%	400
7	Multifunctional contact	98%	97%	–	450
8	Multifunctional contact	98%	–	95%	550
9	Multifunctional contact	98%	97%	95%	800

The first two items in Table 3 are non-contact sensors and the rest are contact sensors. Contact sensors can only measure the parameter at the installation point, whereas non-contact sensors can measure the parameter within a specified radius. The measurement accuracies reported in Table 3 pertain to the sensor installation points. The accuracy of non-contact sensors decreases with distance from the installation point. Although contact sensors can only measure the parameter at the installation point, virtual sensing techniques or expert experience can be used to estimate the parameters in nearby regions (blocks). The estimation accuracy in each block can vary based on the type and location of the sensors, environmental characteristics, and the positioning of heating and cooling equipment. In this case study, the estimation accuracy for blocks near the candidate sensor locations was determined based on expert opinions ($\:{\lambda\:}_{klpt}$). For example, if a type 3 sensor is installed at location 4, the estimated temperature accuracy for blocks 4 to 7 is 94%, 96%, 98%, and 98%, respectively, while for other blocks, it is 0%. Similar determinations were made for other cases, but due to the large volume of data, they cannot be fully reported here.

## Results and experiments

The proposed ILPs were implemented using the IBM^®^ ILOG CPLEX 12.10 package and solved via the OPL interface on a machine running Windows^®^ 10, equipped with 8 GB of RAM and a Core i5 CPU. In this section, the proposed *Leader-Follower Approach* is evaluated through a case study. Two strategies (*Minimum Coverage Assurance Strategy* and *Budget Constraint Strategy*) were introduced in Sect. Methodology to implement this approach. Both strategies are subsequently examined and tested.

**Fig. 3:**
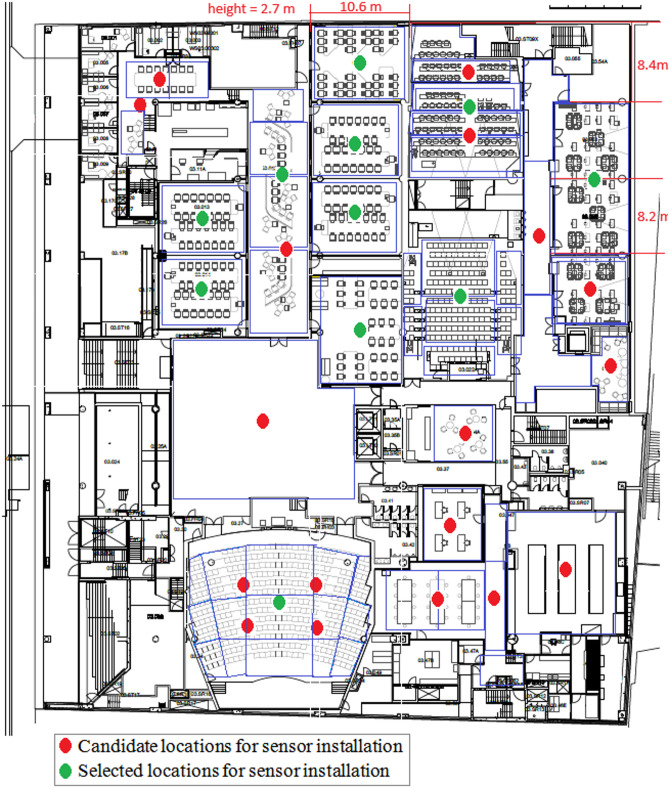
Selected locations for sensor installation in the *Minimum Coverage Assurance Strategy*.

The *Minimum Coverage Assurance Strategy* for implementation requires the desired coverage as an input parameter, which for the case study is considered 60%. The minimum cost required to reach this coverage is $2,550, calculated by the *Cost Optimization Model*. This coverage can be increased to 61.3% without any additional cost by solving the *Coverage Optimization Model*. Therefore, the layout derived from the *Minimum Coverage Assurance Strategy* can cover 61.3% while the cost is $2,250. Figure 3 illustrates the sensor installation locations for this layout. The red circles indicate the candidate locations for sensor installation, while the green circles show the locations where sensors have been installed. A total of 11 sensors were selected for installation at the candidate locations. The type of sensor installed in each location is specified in Table 4.

**Table 4:** The type of sensors installed in candidate locations for *Minimum coverage assurance Strategy*.

Location	3	5	6	7	8	9	10	12	14	16	26
Type	4	4	4	4	4	4	4	7	7	4	7

**Fig. 4:**
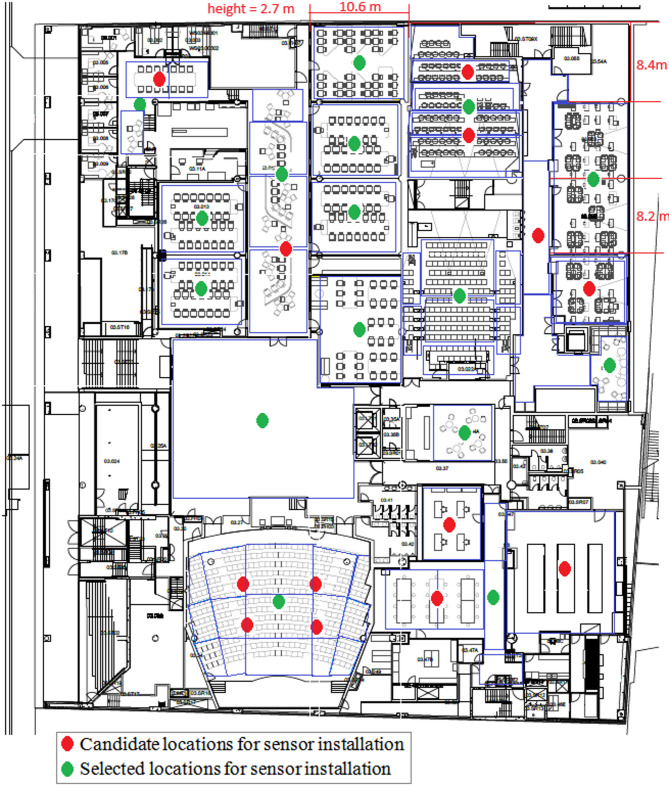
Selected locations for sensor installation in the *Budget Constraint Strategy*.

The budget limits in the *Budget Constraint Strategy* as an input parameter. For the case study, it was set to $4,000. The maximum achievable coverage with this amount is 72.5%, as determined by the *Coverage Optimization Model*. In this scenario, the *Cost Optimization Model* ensures that achieving 72.5% coverage for less than $4,000 is not feasible. Figure 4 shows the obtained optimal layout from the *Budget Constraint Strategy*. The red circles indicate the candidate locations for sensor installation, while the green circles show the locations where sensors have been installed. A total of 16 sensors were selected for installation at the candidate locations. The type of sensor installed in each location is specified in Table 5.

**Table 5:** The type of sensors installed in candidate locations for *Budget constraint Strategy*.

Location	2	3	5	6	7	8	9	10	12	14	16	18	19	20	22	26
Type	4	4	4	4	4	4	4	4	9	7	4	4	4	4	4	9

**Fig. 5:**
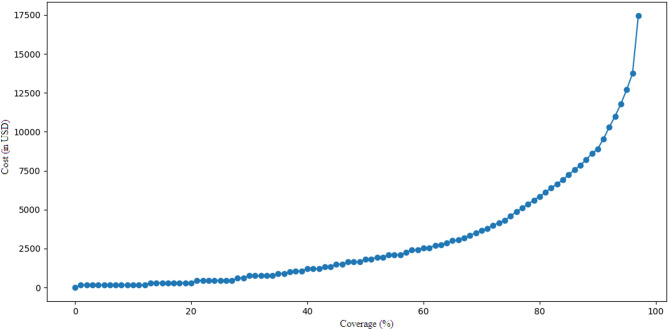
Coverage–cost graph for case study.

By running the *Cost Optimization Model* 100 times, a Coverage-Cost graph was generated, as depicted in Fig. 5. This figure, which integrates Strategies of *Minimum Coverage Assurance* and *Budget Constraint*, provides a valuable opportunity for making optimal decisions regarding sensor placement. For example, the graph shows that a budget of approximately $5,000 is able to achieve 77% coverage. On the other hand, 70% coverage would require $3,500. Also, Fig. 5 shows that the required cost for coverage levels above 80% increases sharply, indicating diminishing returns as the coverage target rises. In contrast, increasing the coverage up to 40% gives less significant cost variations that represent that initial improvements are far more cost-effective.

The following subsections illustrate a sensitivity analysis in order to investigate the dependency of the identified optimal sensor location and deployment scheme on some key parameters. In this regard, it focuses on three critical factors that include: (1) deploying multi-functional sensors, (2) the selection of sensor types, and (3) covering certain areas. This analysis tries to provide more insight into the model’s robustness and flexibility by studying how changes in these variables affect cost, coverage, and the overall effectiveness of the proposed sensor placement method. The findings will help further understand the associated trade-offs involved and guide the decisions on sensor configuration for various operational scenarios.

### Multifunctional sensors

Table 6 shows the impact of considering multifunctional sensors in the proposed method. For instance, achieving 90% and 95% coverage without using multifunctional sensors increases costs by 5.1% and 7.5%, respectively. Therefore, considering multifunctional sensors in the optimization process of the proposed method is able to reduce sensor deployment costs\.

**Table 6:** Exploring the importance of considering multifunctional sensors.

Coverage percentage	Cost without multifunction sensors	Cost with multifunction sensors	Percent Change
50	1800	1800	0%
60	2550	2550	0%
70	3700	3650	1.4%
80	6050	5850	3.4%
85	7450	7250	2.8%
90	9350	8900	5.1%
95	13,650	12,700	7.5%

### Type of sensors

**Fig. 6:**
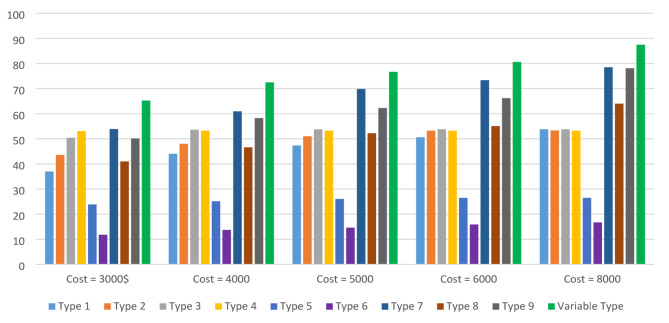
Examining the importance of determining the sensor type in the optimization process.

In the proposed method, sensor type is a decision variable. In order to highlight the importance of this matter, Fig. 6 is drawn in which the sensor type is predefined and the number as well as location of sensors are considered as decision variables. Figure 6 compares coverage levels across different scenarios with fixed costs of $3000, $4000, $5000, $6000, and $8000. For instance, using the sensor of type 7 with a cost of $4000 achieves approximately 61% coverage. In contrast, when sensor type is treated as a decision variable, the same cost yields about 73% coverage. In another comparison, if sensor type is a decision variable, a budget of $3000 can achieve 65% coverage, whereas restricting the sensor type to type 8 requires $8000 to achieve the same coverage. In general, Fig. 6 demonstrates that optimizing sensor type significantly increases coverage compared to predetermined sensor types. Therefore, the simultaneous optimization of sensor type, number and location in the proposed method might give a unique opportunity to cut the costs of the sensor deployment.

### Coverage of specific areas

**Fig. 7:**
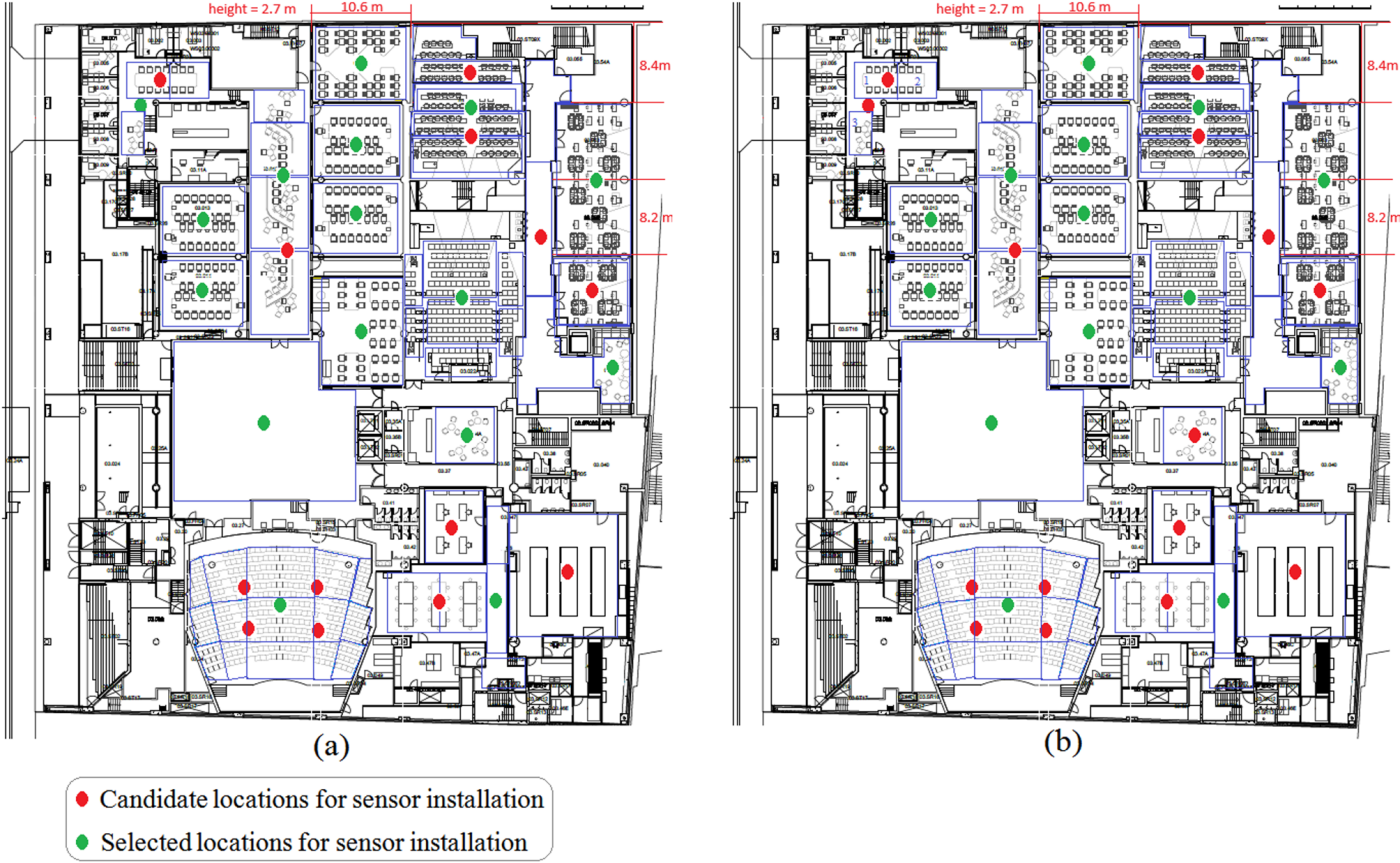
Performance of constraints (7) and (13) in sensor layout: (**a**) Without Constraints and (**b**) With Constraints Applied to Block 33.

In some blocks, it is required that certain parameters be measured. For such requirements constraints (7) and (13) are responsible for the developed models. Figure 7 has been prepared to examine this. Figure 7a shows the optimal sensor layout without applying constraints (7) and (13) and with a budget limit of $4000. In contrast, Fig. 7b provides the optimal deployment plan imposed to restrictions (7) and (13), having applied for Block 33: temperature, humidity, and air velocity should be monitored under the same constraints of budget limitations. Moreover, Table 7 shows the optimal locations and types of sensors installed in these locations .

**Table 7:** Type of sensors installed in candidate locations of Fig. 7.

Location	2	3	5	6	7	8	9	10	12	14	16	18	19	20	22	26
Type (Fig. 6a)	4	4	4	4	4	4	4	4	9	7	4	4	4	4	4	9
Type (Fig. 6b)	–	4	4	4	4	4	4	4	7	7	4	4	–	9	4	9

As shown in Fig. 7; Table 7, in the optimal layout without applying constraints (7) and (13), a type 4 sensor is used at candidate location 20 (Fig. 7a). Consequently, only the temperature in Block 33 is measured. When constraints (7) and (13) are applied to Block 33, a type 9 sensor replaces the type 4 sensor at location 20 (Fig. 7b) to measure temperature, humidity, and air velocity in Block 33. This demonstrates that constraints (7) and (13) can account for coverage requirements in specific areas.

### Performance of the proposed method in multi-story buildings

**Fig. 8:**
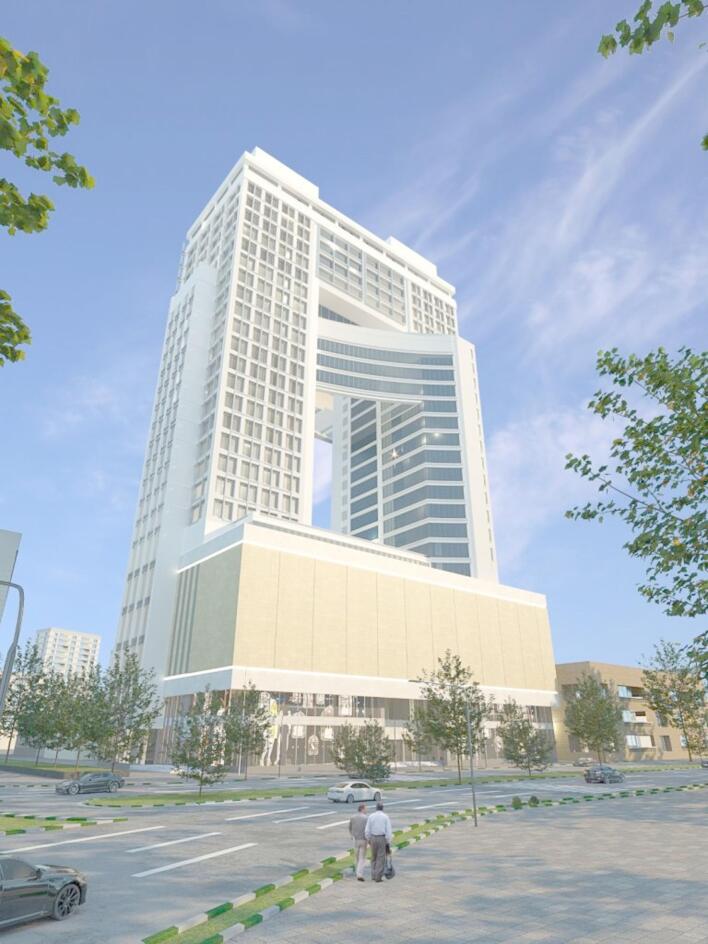
Kooh-Noor Building.

In this section, the performance of the exact ILP models is evaluated for multi-story buildings and compared with an approximate approach based on Grouping of Similar-Usage Floors. The “Kooh Noor” complex in Mashhad, Iran, was selected as a case study. As shown in Fig. 8, the building consists of 35 residential, commercial, and office floors built on a plot of approximately 2,900 m². In this case, 27 floors, including 2 restaurant floors, 2 exhibition floors, 6 commercial floors, and 17 office floors, were considered for sensor placement.

In the Grouping of Similar-Usage Floors approach (approximate approach), one representative floor is selected from each group of similar floors. In the current case study, one floor from the restaurants and exhibitions, one commercial floor, and one office floor were chosen as representatives. The block importance weights and sensor installation costs are scaled according to the number of similar floors; for instance, all block weights and sensor costs in the representative commercial floor are multiplied by six (See Sects. Methodology–Results and Experiments for more details).

Table 8 reports the results obtained from both the exact and approximate approaches for coverage levels of 40%, 50%, 60%, and 70%. At 40% coverage, the exact ILP approach was terminated after 3117 s due to memory limitations on a system with 8 GB RAM. The obtained solution had a gap of 1.27% and a total cost of 6,450$. In contrast, the approximate method reached a solution with a cost of 7,200$ in only 49 s. At 50% coverage, the exact approach achieved the optimal solution of 9,150$ after 307 s, whereas the approximate approach produced a solution with a cost of 9,600$ in just 6 s. At 60% coverage, the exact approach reached the optimal solution with a cost of 13,150$ after 3,923 s, while the approximate method provided a solution with a cost of 13,400$ in 139 s. At 70% coverage, however, the approximate method outperformed the exact ILP model, obtaining a solution with a cost of 19,800$ in only 13 s. In contrast, the exact method was stopped due to memory limitations at a gap of 1.51% and produced a solution with a cost of 20,050$. These results demonstrate that the approximate approach tends to achieve competitive, and in some cases superior, solutions as the coverage level increases.

**Table 8:** Performance of exact and approximate methods in Multi-Story sensor Placement.

Coverage	Method	Runtime (s)	Cost ($)	Gap (%)
40%	Exact	3117	6450	1.27
	Approximate	49	7200	13.05
50%	Exact	307	9150	0
	Approximate	6	9600	4.92
60%	Exact	3923	13,150	0
	Approximate	139	13,400	1.90
70%	Exact	3134	20,050	1.51
	Approximate	13	19,800	0.02

In general, the results in Table 8 show that the approximate approach achieves the solution in less time compared to the exact approach. This difference is mainly attributed to the number of variables and constraints involved. Specifically, the number of constraints and variables in the exact approach are 9,212,601 and 9,215,520, respectively, whereas in the approximate approach they are reduced to 173,361 and 173,760. Nevertheless, the exact method remains more reliable for ensuring accuracy. In some cases, the exact method may encounter memory limitations, which can typically be overcome by employing higher-capacity hardware. However, when such resources are not available, the similar-use floor grouping approach can serve as a practical and efficient alternative. In summary, the results of this section demonstrate that the methods proposed in this study remain effective and scalable for real-world applications as the number of floors increases.

##  Conclusion

In this paper, two ILP models were introduced to optimally solve the building climate control sensor layout problem optimally. One model focuses on cost optimization, and the other on coverage optimization. A Leader–Follower Approach, or bi-level programming, is adopted to optimize both coverage and cost simultaneously, with the advantage of obtaining the optimal solution in a single iteration.

The results reveal that the proposed method can determine the optimal number, type, and location of sensors with minimal execution time. The optimization process aims to minimize the cost of purchasing and installing sensors while maximizing coverage and measurement accuracy. The precision of virtual sensing is treated as an input parameter in the proposed method. This process prioritizes reducing sensor deployment costs while improving coverage and measurement accuracy. By considering factors such as multifunctional sensor types, sensor placement, and coverage requirements for specific areas, the proposed method offers a balanced approach to sensor layout optimization. Furthermore, the integration of virtual sensing precision as an input parameter is a key feature, enabling the method to adapt to different environmental configurations.

## Model limitations and future directions

The proposed method focuses on determining the optimal location and type of sensors required for buildings. However, it has certain limitations that can be addressed in future research:

- **Dependence on virtual sensing accuracy**: In this study, expert judgment was employed to estimate the accuracy of virtual sensing. Integrating the proposed method with data-driven virtual sensing approaches in future work could help mitigate potential biases.
- **Block importance estimation**: The current approach assigns sensor locations and types based on the importance of each block, which was determined through expert judgment. Developing systematic methods for quantifying block importance and integrating them into the proposed framework could provide a more objective basis for decision-making.
- **Dynamic building conditions**: Sensor deployment can be influenced by seasonal variations, changes in space utilization, and fluctuating occupancy patterns. Developing the proposed method to account for dynamic environments may reduce the need for frequent sensor relocation and adjustments, ultimately lowering deployment costs.

## Data Availability

The source code, datasets, and supplementary materials developed for this study are publicly available in the GitHub repository associated with this paper. https://github.com/smartconstructiongroup/Sensor-layout.
